# The effects of physical activity on self-esteem in older adults: a systematic review

**DOI:** 10.3389/fpubh.2025.1595087

**Published:** 2025-06-10

**Authors:** Stefan Mijalković, Stefan Stojanović, Ana Lilić, Tamara Ilić, İsmail İlbak, Krzysztof Kasicki, Patryk Niewczas-Czarny, Tadeusz Ambroży, Łukasz Rydzik

**Affiliations:** ^1^Faculty of Sport and Physical Education, University of Niš, Niš, Serbia; ^2^Institute of Health Sciences, İnönü University, Malatya, Türkiye; ^3^Department of Physiotherapy, Faculty of Health Sciences, Collegium Medicum, Andrzej Frycz-Modrzejewski Krakow University, Kraków, Poland; ^4^Institute of Physical Culture Sciences, College of Medical Sciences, University of Rzeszów, Rzeszów, Poland; ^5^Institute of Sports Sciences, University of Physical Culture, Kraków, Poland

**Keywords:** exercise, physical activity, self-esteem, training, aging

## Abstract

**Background:**

The aim of this systematic review was to evaluate the impact of physical activity on self-esteem levels in older adults.

**Methods:**

This review was conducted in accordance with PRISMA 2020 guidelines. PubMed and Web of Science databases were searched using the following terms (in English): *(physical activity OR physical exercise OR training) AND (self-esteem OR self-confidence OR self-respect) AND (male OR female) AND older adults*.

**Results:**

Out of 3,732 identified publications, after removing duplicates and initial screening, 17 studies were ultimately included in the analysis (total of 1,820 participants). All included studies showed a statistically significant positive relationship between regular physical activity and higher self-esteem in older adults. Particularly beneficial effects were observed for aerobic training (e.g., walking, jogging, cycling), yoga sessions, and exercises targeting strength, endurance, and coordination development.

**Conclusion:**

Regular participation in activities such as walking, yoga, and moderate-intensity exercises positively influences self-esteem in older adults, making physical activity an important factor for psychological well-being in later life.

**Systematic review registration:**

The systematic review was registered in the PROSPERO database and is available under the ID number: CRD420251011376, https://www.crd.york.ac.uk/PROSPERO/view/CRD420251011376.

## 1 Introduction

Physical activity refers to any physical movement produced by skeletal muscles that requires energy expenditure (1). Specifically, engaging in physical activity is one of the most important factors in improving and maintaining overall health by reducing decline and sustaining optimal levels of functional and motor abilities (2–5). Moreover, regular physical activity is intrinsically linked to healthy lifestyle habits and can lead to a reduction in the risk of morbidity and premature mortality, while also enhancing the overall quality of life for individuals (6–10).

Furthermore, it has been established that physical activity can counteract many comorbidities associated with aging that affect the musculoskeletal, circulatory and cardiovascular systems (11). Additionally, engaging in exercises designed to improve coordination, strength, and flexibility enhances self-efficacy, boosts self-esteem, promotes overall well-being and reduces anxiety in older adults who do not have health issues (12, 13). Self-esteem is considered one of the key aspects of psychological well-being and represents an essential factor for a successful and fulfilling life in older adults (14). Specifically, some research has demonstrated that self-esteem is significantly influenced by physical activity in older adults (15, 16, 43). Additionally, self-esteem represents an individual’s overall evaluation of their own worth, encompassing feelings of self-respect and self-acceptance, and is considered a crucial aspect of psychological well-being (14). In the reviewed studies, different self-esteem measurement scales were employed, including the Rosenberg Self-Esteem Scale for global self-esteem and other instruments assessing domain-specific dimensions such as physical or social self-esteem (17, 18). Given the heterogeneity of these tools, interpretations of the effects of physical activity should carefully consider the type of self-esteem evaluated (17).

It can be said that depression, loneliness, anxiety, alienation and insufficient physical activity or a complete lack of it contributes to low self-esteem (6, 19–21). Unwanted loneliness, resulting from a gap between desired and actual social connections is a growing public health concern, particularly for older adults (22). It is linked to lower self-esteem, depression, and reduced motivation for health behaviors like physical activity (23–25). Physical activity, especially in community settings, helps reduce loneliness and improve well-being (26). Group activities, like walking clubs, foster social interaction and community engagement, which are vital for strengthening social ties and promoting active aging (1).

Specifically, inactivity in older adults can lead to significant physical and psychological complications, one of which is a gradual decline of self-esteem (21, 27). Many research articles have explored the positive effects of physical activity on self-esteem in older adults (23, 25, 28–30). Namely, jogging and walking lead to higher self-esteem, improved sleep quality, better mood, increased life satisfaction and reduced stress in older adults (25, 29). Moreover, high self-esteem is associated with engaging in exercises that enhance strength, coordination, flexibility, endurance, and balance (23). This connection contributes to a reduced in depression, anxiety, and the likelihood of injuries among older adults (23, 28, 30).

There is a considerable necessity to conduct a systematic review that will comprehensively synthesize available evidence, identify potential moderators of these effects, and offer practical recommendations for designing effective physical activity interventions aimed at improving self-esteem in this population. Previous studies have varied in terms of intervention type, duration, and assessment methods, making it difficult to draw unified conclusions about the consistency and strength of these effects (23, 25, 28). Addressing this issue could help healthcare professionals, gerontologists, and policymakers create evidence-based programs focused on promoting psychological well-being and quality of life in older adults. Given all of the above, the aim of this research was to summarize and analyze previous studies to determine the effects of physical activity on self-esteem in older adults.

## 2 Methods

### 2.1 Literature search

The research was analyzed and searched in accordance with PRISMA (Preferred Reporting Items for Systematic Reviews and Meta-Analyses) standards (31). In this systematic review, PubMed and Web of Science were selected as primary databases for literature search due to their broad multidisciplinary coverage and high indexing standards, particularly for health sciences, physical activity, and public health-related studies. These databases were considered most relevant as the primary focus of this review was on the health-related effects of physical activity on self-esteem in older adults, an area extensively covered in biomedical and public health journals. The systematic review was registered in the PROSPERO database and is available under the ID number: CRD420251011376. Additionally, the following terms were used when searching electronic databases: (physical activity OR physical exercise OR training) AND (self-esteem OR self-confidence OR self-respect) AND (male OR female) AND older adults. The systematic review project started on 01/11/2024 and ended on 01/03/2025. The articles were screened by two authors (K.K and I.I.). The search strategy for the research is presented in Table 1.

**Table 1:** PICO criteria.

	Inclusion criteria	Exclusion criteria
Population	Older adults of both genders, aged 60 years and older, healthy participants	Participants under the age of 60; populations with health conditions, chronic diseases, or disabilities
Intervention	Physical activity	No physical activity as an intervention
Comparison	Control group, intervention, treatment	No control group, no intervention, no treatment
Outcome	Self-esteem (improvement in self-esteem in older adults)	No data on self-esteem in older adults

**Figure 1:**
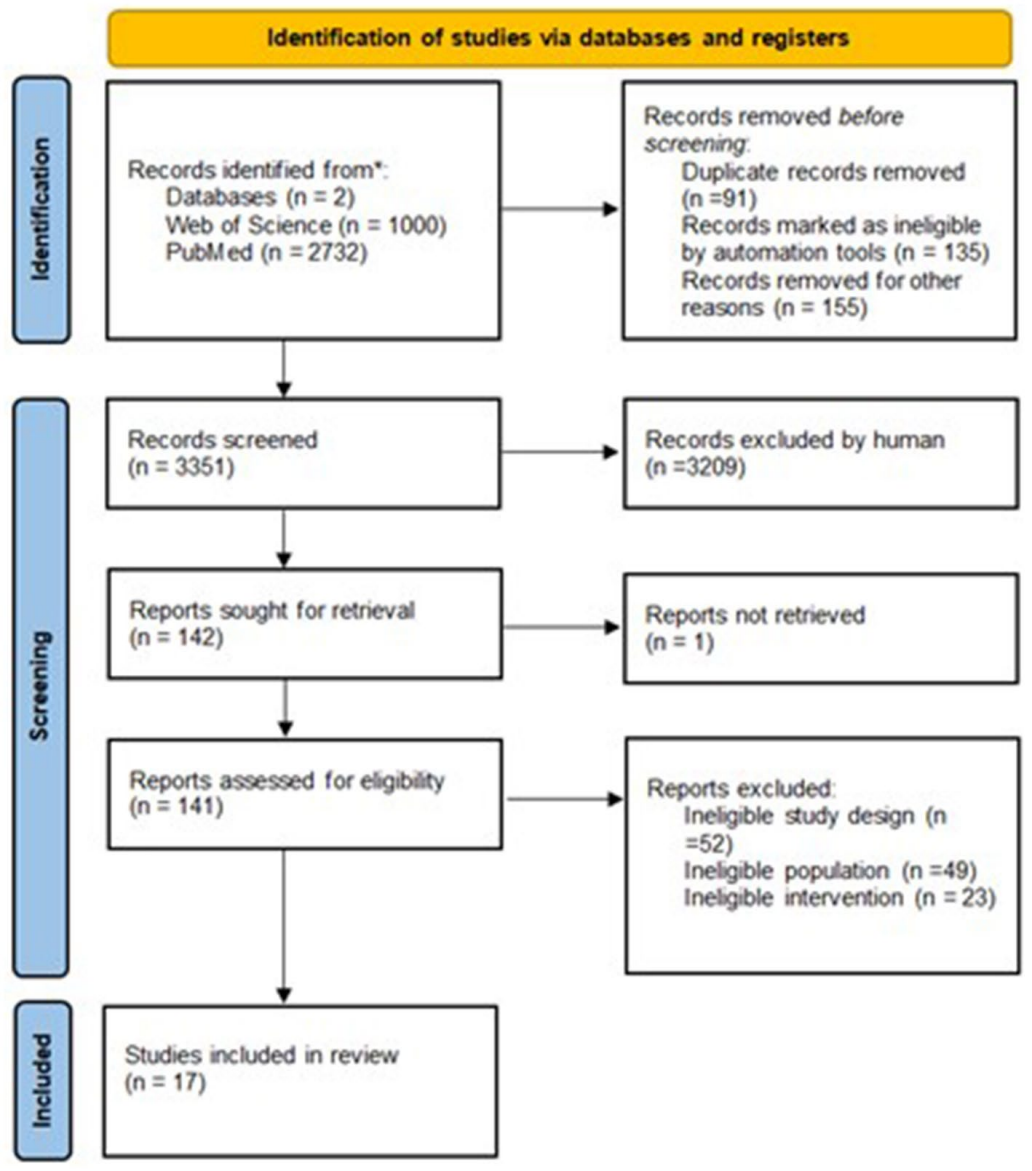
PRISMA flowchart.

Specifically, descriptive methods, analysis, and synthesis were used to develop this research. The included research was selected based on titles, keywords, abstracts, and primarily based on full texts. The research was carefully chosen, and the selected research met the inclusion criteria for the final analysis. The process of collecting research is illustrated in Figure 1.

### 2.2 Inclusion criteria

In order for the research to be included in the final analysis, it had to meet the following criteria. The first criterion was that the research investigated the effects of physical activity on self-esteem. Consequently, studies that did not address this topic were eliminated. The second criterion was that the studies involved older adults of both genders (aged 60 and older). The third criterion was that the participants were healthy. The fourth criterion was that the studies were fully published in English. Finally, the fifth exclusion criterion required the studies to be of an original type.

### 2.3 Exclusion criteria

Studies were excluded from the final analysis if they did not investigate the effects of physical activity on self-esteem in older adults, involved participants under the age of 60, or focused on populations with health conditions, chronic illnesses, or disabilities that could affect physical activity or self-esteem outcomes. Additionally, studies that were not published in English or were systematic reviews, meta-analyses, or secondary analyses of existing datasets were excluded. Only original research articles, including randomized controlled trials, cohort studies, and cross-sectional studies, were considered eligible for inclusion. The PICO (Population, Intervention, Comparison, Outcome) criteria are presented in Table 1.

### 2.4 Data extraction

Data from the articles included in the systematic review were collected using a previously prepared, standardized data extraction form. Three authors (K.K., T.A., and Ł.R.) performed the extraction of information, including:

1. Bibliographic data: author(s), year of publication, country, and journal.
2. Participant characteristics: number of participants, age, sex, health status, inclusion and exclusion criteria.
3. Intervention/type of physical activity: a detailed description of the intervention (e.g., type of exercises, intensity, duration, frequency of sessions).
4. Comparison/control: if there was a control group, a description of its characteristics and the type of activity undertaken (or lack thereof).
5. Main baseline measures and assessment tools: a self-esteem assessment scale (e.g., the Rosenberg Scale), and any other physical and mental health indicators.
6. Outcomes: key quantitative or qualitative results, statistical significance, effect size (if provided).
7. Additional information: funding, conflict of interests, authors’ conclusions, and study limitations.

Any uncertainties were resolved through discussion, and if consensus could not be reached, the deciding voice belonged to the fourth author responsible for the analysis (P.N-C.). In the case of missing data, the authors attempted to contact the authors of the original studies.

### 2.5 Effect measures

Because the review primarily included quantitative studies concerning self-esteem levels, effect measures (if available in the primary publications) such as mean and standard deviation (or standard error) for the main outcome, as well as statistical significance (*p*-value), were adopted. Other effect measures were not included in the review due to its narrative nature and the lack of sufficient data in the included publications.

### 2.6 Synthesis methods

The studies were grouped according to the similarity of interventions (e.g., aerobic exercise, yoga, strength training) and comparable outcome measures (e.g., Rosenberg scale vs. other scales). The completeness of the mean and standard deviation data for each group and time point was verified. In the case of inconsistencies in reporting methods, a standardized approach was applied wherever the original data permitted.

Given the diverse intervention methods and outcome measures, a descriptive (narrative) synthesis was employed. Due to the heterogeneity of study designs (participant characteristics, different types and durations of interventions), no formal statistical assessment of heterogeneity was performed. If the dataset is expanded or new studies become available, subgroup analyses may be conducted.

### 2.7 Risk of bias assessment

The quality and suitability of the research for the final analysis were assessed by three independent authors (S.M. and S.S.). A web tool called “Rayyan” was used for blind review (32). In cases of disagreement in the assessment of bias risk, the data was reviewed by a third author (A.L.), who made the final decision.

## 3 Results

### 3.1 Quality of research

The results of the quality assessment of the research were calculated using the total number of research included in the quantitative synthesis and the scores that each research received on the PEDro scale (33). The results of the PEDro scale are presented in Table 2.

**Table 2:** Results of the PEDro scale.

	Criterion											
Research	1	2	3	4	5	6	7	8	9	10	11	**∑**
Escolar Chua et al. (28)	Y	Y	Y	Y	N	N	N	Y	Y	Y	Y	7
Kleinert et al. (29)	Y	Y	Y	Y	N	N	N	Y	N	Y	Y	6
Olsen et al. (16)	Y	Y	Y	Y	N	N	N	N	Y	Y	Y	6
Awick et al. (17)	Y	Y	Y	Y	N	N	N	Y	Y	Y	Y	7
Jung et al. (41)	Y	N	N	Y	N	N	Y	Y	Y	Y	Y	6
Moral-García et al. (42)	Y	Y	Y	Y	N	N	N	Y	Y	Y	Y	7
Amesberger et al. (38)	Y	N	N	Y	N	N	N	Y	Y	Y	Y	5
Finkenzeller et al. (39)	Y	N	N	Y	N	N	Y	Y	Y	Y	N	5
Fougner et al. (15)	Y	Y	Y	Y	N	N	N	Y	Y	Y	Y	7
Knapik et al. (40)	Y	N	N	Y	N	N	N	Y	Y	Y	Y	5
Borbón-Castro et al. (23)	Y	N	N	N	N	N	Y	Y	Y	Y	Y	5
Guimarães et al. (35)	Y	Y	Y	Y	N	N	N	Y	Y	Y	Y	7
Goh et al. (36)	Y	N	N	Y	Y	N	Y	Y	Y	Y	Y	6
Li et al. (30)	Y	Y	Y	Y	N	N	N	Y	Y	Y	Y	7
Grajek et al. (34)	Y	Y	Y	Y	N	N	N	Y	Y	Y	Y	7
Nugraha et al. (18)	Y	N	N	N	N	N	Y	Y	Y	Y	Y	5
Toros et al. (25)	Y	Y	Y	Y	N	N	N	Y	Y	Y	Y	7

1—eligibility criteria; 2—random allocation; 3—concealed allocation; 4—baseline comparability; 5—blind subject; 6—blind clinician; 7—blind assessor; 8—adequate follow-up; 9—intention-to-treat analysis; 10—between-group analysis; 11—point estimates and variability; Y—criterion is satisfied; N—criterion is not satisfied; ∑—total awarded points.

### 3.2 Selection and characteristics of the research

A total of 3,732 research articles were identified through the search of electronic databases. Three thousand three hundred fifty-one research articles were screened after duplicates were removed. A total of 3,209 research were rejected due to inclusion criteria. In total, 142 research articles were selected and assessed for suitability. Since one research did not have the full text available, 141 research articles were assessed for suitability. The full texts of the remaining research articles were thoroughly reviewed, after which 17 research articles were included in the final analysis. The studies included in the final analysis varied in their design and methods. Studies were experimental (e.g., randomized controlled trials). The physical activity interventions included in the studies were primarily aerobic exercises (e.g., walking, jogging), with some studies also incorporating resistance training and combined physical activity programs. The primary outcome index across all studies was self-esteem, assessed using various standardized scales and questionnaires designed to measure changes in participants’ self-perception and confidence.

Table 3 presents an overview of the analyzed research articles. The table contains information on the first author and year of publication, the participant sample (number, gender, and age), information on the assessment of physical activity, self-esteem assessment, and the findings of the research.

**Table 3:** Overview of the research.

First author and year of publication	Country	Participant sample		Physical activity	Self-esteem	Research findings
		Number	Age			
Escolar Chua et al. (28)	Philippines	T = 40 М = 16 F = 24	60–65	30–40 min PA (SDE, VO_2max_)	Rosenberg scale	PА↔SE
Kleinert et al. (29)	Germany	T = 66 М = 20 F = 46	65.4 ± 4.3	45 min PА (walking)	Physical Self-Description Questionnaire	PА↔SE
Olsen et al. (16)	Norway	T = 8 М = 1 F = 7	69–96	50–60 min PА (SDE, BDE)	Semi-structured interview	PА↔SE
Awick et al. (17)	United States of America	T = 307 М = 71 F = 236	71.0 ± 5.1	30 min PА (FDE, SDE, BDE)	Rosenberg scale	PА↔SE
Jung et al. (41)	South Korea	T = 12 М = 7 F = 5	65–85	30 min LI PА	Rosenberg scale	PА↔SE
Moral-García et al. (42)	Spain	T = 168 M = 39 F = 129	73.6 ± 5.8	120 min LI PА	Rosenberg scale	PА↔SE
Amesberger et al. (38)	Austria	T = 22 М = 9 F = 13	65–70	PSS, VO_2max_	Frankfurter Self-Perception Scale	PА↔SE
Finkenzeller et al. (39)	Austria	T = 22 М = 9 F = 13	69.0 ± 2.0	RPE6, RPE20 (EDE, SDE)	General Mood Survey adapted by Hackford and Schlattmann	PА↔SE
Fougner et al. (15)	Norway	*F* = 16	70–85	60 min MI PA (EDE, BDE)	Semi-structured interview	PА↔SE
Knapik et al. (40)	Poland	T = 123 М = 39 F = 84	68.5 ± 6.2	SFT	SF-36 questionnaire	PА↔SE
Borbón-Castro et al. (23)	Mexico	T = 45 М = 10 F = 35	67.2 ± 5.7	60 min PА (SDE, VDE, FDE, CDE)	Rosenberg scale	PА↔SE
Guimarães et al. (35)	Brazil	T = 36	60.5 ± 2.9	60 min PА (yoga)	Steglich questionnaire	PA↔SE
Goh et al. (36)	South Korea	*F* = 20	80–90	50 min LI PА	Rosenberg, Jeon and Lee scale	PА↔SE
Li et al. (30)	China	T = 80 М = 32 F = 48	68.6 ± 5.9	SDE, BDE, CDE	Rosenberg scale	PА↔SE
Grajek et al. (34)	Poland	*F* = 600	60–70	IGS	Rosenberg scale	PА↔SE
Nugraha et al. (18)	Indonesia	T = 40	60–74	cycling	Self-Esteem Rating Scale	PA↔SE
Toros et al. (25)	Türkiye	M = 215	67.5 ± 7.8	45 min PА (jogging)	Coopersmith Self-Esteem Inventory	PA↔SE

↔ - statistically significant effects (*p* < 0.05). BDE, balance development exercises; CDE, coordination development exercises; EDE, endurance development exercises; F, female participants; FDE, flexibility development exercises; IGS, individualized gymnastics for seniors; LI, low-intensity physical activity; М, male participants; MI, moderate-intensity physical activity; PA, physical activity; PSS, Physical Self-Perception Scale; RPE20, Rate of Perceived Exertion Scale to Exhaustion; RPE6, Rate of Perceived Exertion Scale; SDE, strength development exercises; SE, self-esteem; SFT, Senior Fitness Test; T, Total number of participants; VDE, velocity development exercises; VO_2max_, maximal oxygen consumption.

Seventeen research articles were analyzed in Table 3. It can be concluded that all research demonstrated statistically significant effects of physical activity on self-esteem in older adults. Specifically, participants who were more physically active had higher self-esteem than those who did not engage in any form of physical activity. It can be noted that the oldest research was published in 2014 (28), while the most recent research dates back to 2023 (25). The research articles were conducted with participants of both genders. In two studies, the participants were exclusively female (15, 34), while in one study, the participants were exclusively male (25). Overall, female participants predominated across the studies. The youngest participants were 60 years old (28, 35), while the oldest were 96 years old (16). The smallest number of participants was eight (16), while the largest number of participants was 600 (34). In total, it can be stated that the overall number of participants across all research was 1820.

## 4 Discussion

The aim of this research was to summarize and analyze previous studies to determine the effects of physical activity on self-esteem in older adults. Analysis of previous research revealed statistically significant effects of physical activity on self-esteem in older adults. Analysis of previous research determined statistically significant effects of physical activity on self-esteem in this population. The reviewed studies, published between 2014 and 2023, included participants aged 60 to 96 years and incorporated various physical activities such as walking, jogging, yoga, gymnastics for all, and exercises targeting basic motor abilities. These activities consistently demonstrated benefits for psychological well-being, including enhanced self-esteem, reduced depression, and increased life satisfaction.

Although positive self-esteem is broadly acknowledged as being associated with overall well-being and enhanced psychological functioning (15, 36, 43), these general associations are distinguished from the specific findings derived from the current review. Specifically, positive self-esteem is reflected as an aspect of psychological health, but it can also be seen as a protective factor that contributes to positive social behavior and better overall health status (37). It has been established that walking, jogging and cycling can greatly contribute to higher self-esteem in older adults (18, 25, 29). The findings indicate that engaging in physical activity is crucial for older adults to enhance their self-esteem, quality of life and sleep, while also reducing anxiety, depression and everyday stress (18, 25). Furthermore, the most significant effects of physical activity on psychological parameters in older adults are generally observed in the younger older adults group, specifically those aged 60–69 (25, 29). This age group demonstrates more substantial effects on mental health, self-esteem, and life satisfaction compared to individuals aged 70 and older, in whom the effects are less pronounced or more stable (15). As for gender differences, no significant effects were found; one study exclusively involved male participants (25), while the remaining two did not report any notable gender-related effects (18, 29).

Additionally, it has also been shown that older adults who practice yoga and individualized gymnastics for all have more positive self-esteem and self-confidence (34, 35). Namely, practicing yoga is a combination of psychological self-control techniques and specific body movements that can help practitioners reduce stress and slow down the aging process, leading to more positive self-esteem (35). On the other hand, physical activity in the form of gymnastics for all improves psychological and physical functions and reduces the risk of chronic diseases in older adults (34). It can be stated that younger participants demonstrated more pronounced positive effects compared to older participants (34, 35). Additionally, although gender differences were not explicitly analyzed, it can be inferred that women experienced greater benefits in terms of self-esteem and stress reduction, while men achieved better results in physical activity outcomes (34, 35). It is recommended that older adults engage in yoga, running, walking or gymnastics for all to enhance their physical and psychological abilities, thereby significantly improving their quality of life.

Furthermore, it has been established that older adults who engage in exercises for the development of basic motor abilities (strength, speed, endurance, flexibility, coordination and balance) have more positive self-esteem than physically inactive individuals (15–17, 23, 28, 30, 38, 39, 43). Functional conditioning and overall physical fitness in older adults have been shown to be reliable predictors of self-esteem (17) (Ferreira et al., 2013). Younger older adults (60–69 years) typically show more pronounced improvements in self-esteem and physical function compared to those over 70, likely due to better physical fitness (17, 38). Although gender differences are not always explicitly analyzed, research indicates that women often experience more significant improvements in self-esteem and emotional well-being (23, 38).

Participating in moderate-intensity exercises aimed at developing basic motor abilities plays a significant role in how older adults perceive and experience their body image, which positively affects their self-esteem and self-confidence (15, 39). Furthermore, it has been established that older adults who engage in regular physical activity have more positive self-esteem and improved overall health compared to older adults who spend their leisure time in a sedentary manner (16, 23, 30). It is recommended that older adults engage in regular physical activity to improve their self-esteem and health status, while also reducing the risk of depression and anxiety (28). Younger older adults of both sexes may experience greater benefits in terms of self-esteem from physical activity compared to their older counterparts, particularly men and individuals over the age of 80 (15, 23). This highlights the importance of promoting regular exercise participation, especially within the oldest age groups and among older men, who may be at increased risk of experiencing lower self-esteem due to physical inactivity.

Physical activity is closely related to positive well-being in older adults through feelings of self-esteem and a sense of purpose in life (36). Namely, self-esteem can be seen as a promoter of psychological, physical and social well-being, as it encourages participation in cognitive and social activities and slows down the aging process (40). This fact confirms the existence of the effect of physical activity on self-esteem in older adults (36, 40). Regular low to moderate-intensity physical activity in older adults is linked to higher self-esteem, better physical functioning, reduced risk of dependency on others, and a lower likelihood of depression (41, 42). It can be said that physically active older adults have greater life satisfaction, self-esteem, and better independence compared to physically inactive individuals. Therefore, engaging in physical activity in later life is recommended.

The main strength of this study is that it systematically summarized evidence on the effects of physical activity on self-esteem in older adults, covering research published between 2014 and 2023. It included an age range of participants (60–96 years) and various types of physical activity interventions, such as yoga, walking, jogging, gymnastics for all, and exercises for basic motor abilities. This provided a clearer insight into which activities most effectively improve self-esteem in older adults. Additionally, the study emphasized the importance of physical activity not only for self-esteem but also for enhancing life satisfaction, independence, and overall well-being in later life.

A limitation of this systematic review is the lack of standardization in the tools used to measure self-esteem. The included studies used various scales such as the Rosenberg Self-Esteem Scale, the Physical Self-Description Questionnaire, the Coopersmith Self-Esteem Inventory, and locally adapted instruments. Differences in theoretical constructs and measurement scopes across these tools may have contributed to result heterogeneity. The other limitation of this study is reflected in the small number of published scientific papers that met all the inclusion criteria for participation in this systematic review. In many studies, participants were not in good health due to their age. Additionally, female participants predominated in the implementation of this research. A recommendation for future research would be to examine the effects of physical activity on self-esteem in older male adults, as well as to investigate whether these effects differ according to sex and specific age groups.

## 5 Conclusion

Based on the findings of this systematic review, it was concluded that physical activity had statistically significant effects on self-esteem among older adults. In particular, engaging in physical activity positively influenced self-esteem, enhanced sleep quality, boosted mood and improved overall health status. Physical activities such as walking, jogging, yoga, gymnastics for all, exercises for the development of basic motor abilities and moderate-intensity exercises significantly influence self-esteem in older adults. Therefore, participating in physical activity in later life is crucial for enhancing self-esteem.

## Data Availability

The original contributions presented in the study are included in the article/supplementary material, further inquiries can be directed to the corresponding authors.
